# Supplementary material for: Essential omega-3 fatty acids tune microglial phagocytosis of synaptic elements in the mouse developing brain
Source: Nat Commun. 2020 Nov 30;11:6133. doi: 10.1038/s41467-020-19861-z (PMC7704669; doi:10.1038/s41467-020-19861-z)
Supplement: Supplementary file 1 — Supplementary Information [file 41467_2020_19861_MOESM1_ESM.pdf]

SUPPLEMENTARY INFORMATION

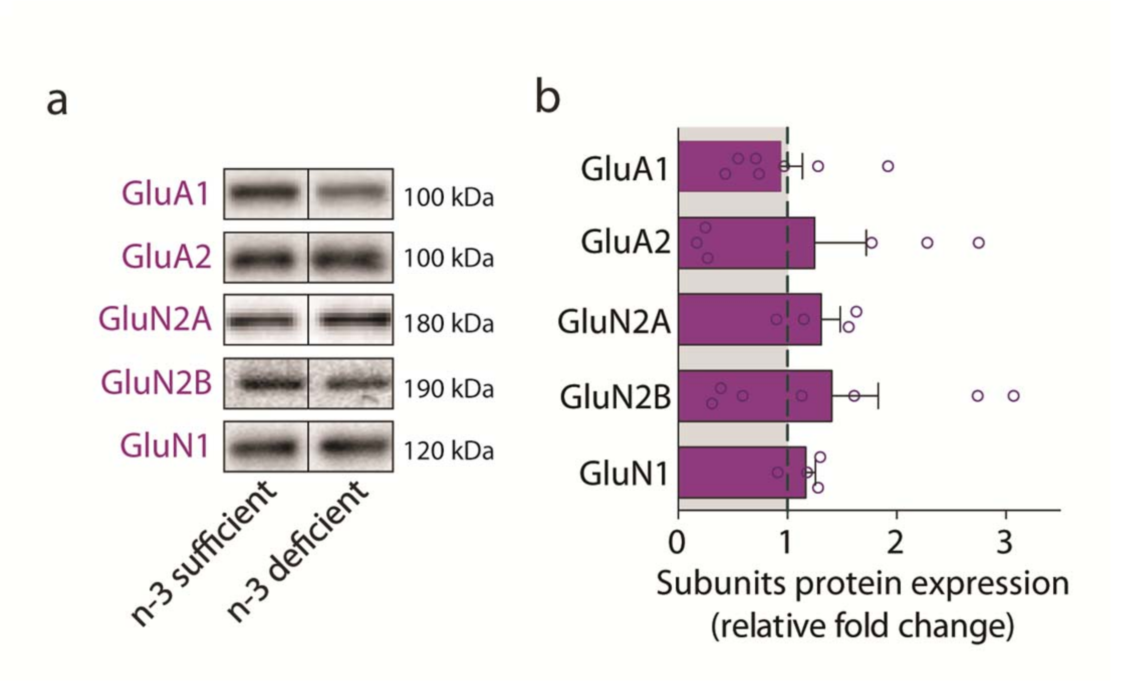

Supplementary Figure 1. Effect of maternal n-3 PUFA deficiency on ionotropic glutamatergic receptor subunits expression level. a. Representative Western blots for GluA1, GluA2, GluN2A, GluN2B and GluN1 subunits from 2 independent experiments. **b.** Quantification of ionotropic glutamatergic receptor protein expression in n-3 deficient mice relative to n-3 sufficient mice. Means ± SEM; n=4-7 mice per group. Two-tailed unpaired Student's t-test, t=1.226, p=0.2597, GluA1; t=0.7894, p=0.4452, GluA2; t=1.413, p=0.201, GluN2A; t=0.417, p=0.6864, GluN2B; t=0.2475, p=0.8087, GluN1. Source data are provided as a Source Data file.

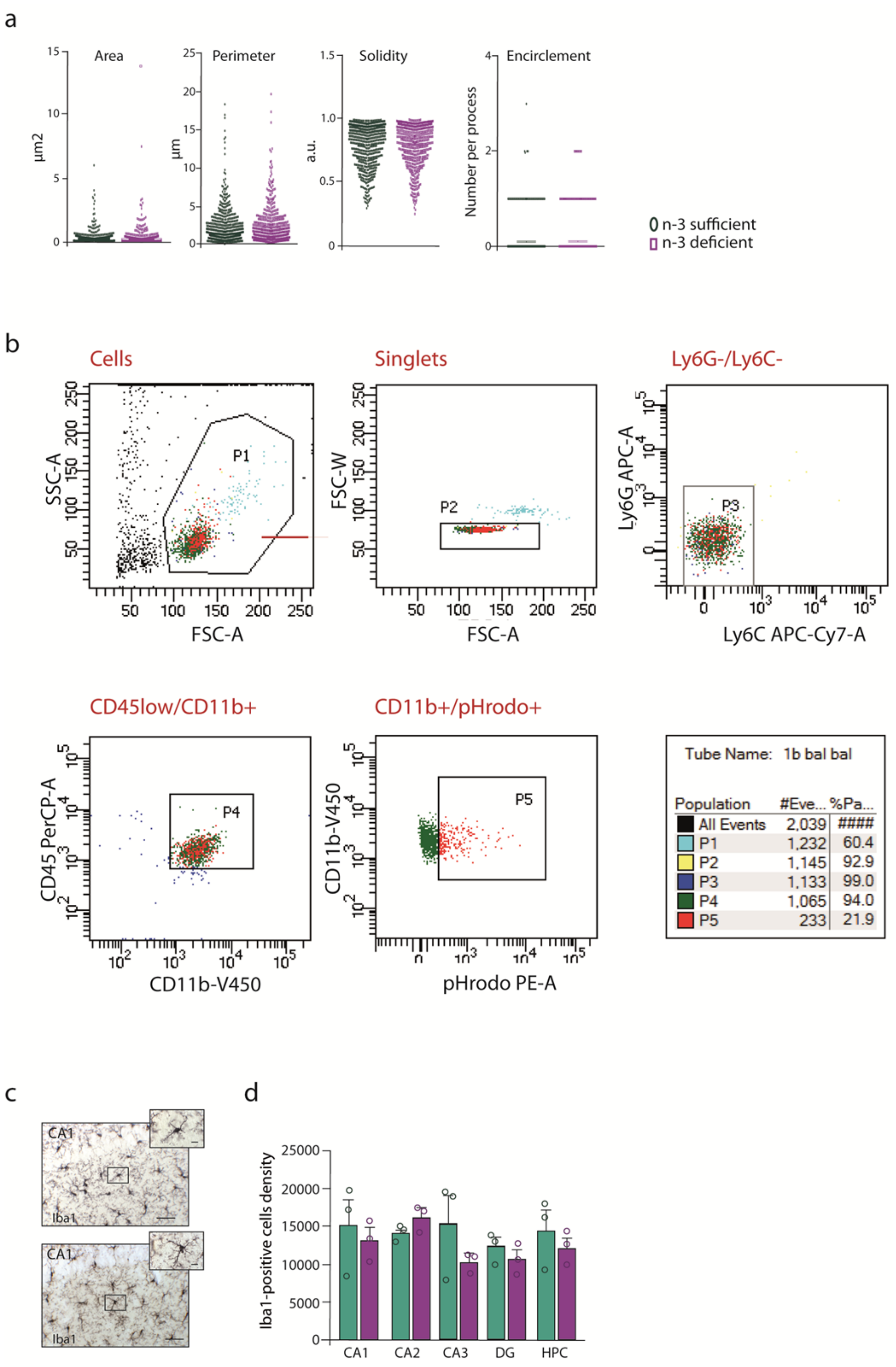

Supplementary Figure 2. Maternal n-3 PUFA deficiency does not modify microglial fine morphology and density. a. EM analysis of the fine morphology of Iba1 positive microglial processes (area, perimeter, solidity i.e. spatial density, and encirclement i.e. ability to enwrap synapses). Means ± SEM; n=627-270 processes per group. Two-tailed unpaired Student's t-test, t=0.9621, p=0.3362, area; t=0.82, p=0.412, perimeter; t=0.487, p=0.6264, solidity;

t=0.3607, p=0.7184, encirclement. **b.** Gating strategy for ex vivo microglia phagocytosis experiments corresponding to Figure 2, 3 and 7. Figure shows representative FACS plots of gates P1 (Cells), P2 (Single cells), P3 (Ly6G-ve/Ly6G-ve to gate out neutrophil and monocyte populations), P4 (CD45low/CD11b+ve Microglia), P5 (pHrodo +ve synaptosomes phagocytosed by microglia). **c.** Representative images of Iba1 immunostained microglial cells in the CA1 region of the hippocampus from 1 independent experiment. Scale bar=100µm. Scale bar insert=10µm. **d.** Stereological counting of microglial cells density in the hippocampus of n-3 deficient and n-3 sufficient mice. Means ± SEM; n=3 mice per group. Two-tailed unpaired Student's t-test, t=0.47, p=0.66, CA1; t=1.85, p=0.14, CA2; t=1.34, p=0.25, CA3; t=0.97, p=0.38, DG; t=0.77, p=0.48, whole hippocampus. Source data are provided as a Source Data file.

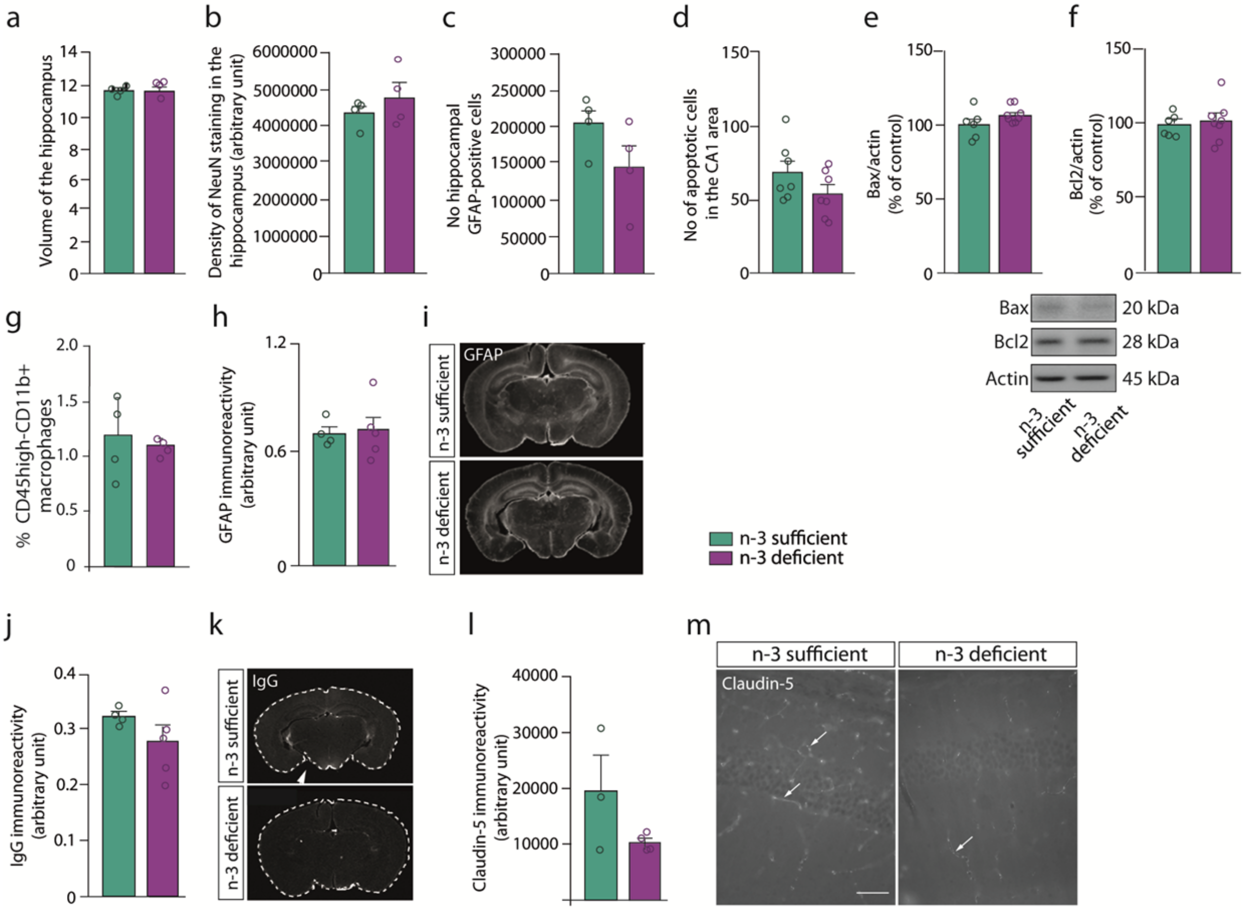

Supplementary Figure 3. Maternal n-3 PUFA deficiency does not modify hippocampus volume and cell content, apoptosis, macrophages infiltration and BBB integrity. a. Stereological measurements of hippocampal volume in n-3 deficient and n-3 sufficient mice. Means ± SEM; n=4 mice per group. Two-tailed unpaired Student's t-test, t=1.012, p=0.35. **b-c.** Quantification of NeuN immunostaining density (B) and number of GFAP-positive cells (**c**) within the hippocampus of n-3 sufficient and n-3 deficient mice cells. Means ± SEM; n=4 mice per group. Two-tailed unpaired Student's t-test, p=0.36, NeuN; t=1.64, p=0.15, GFAP. **d.** Quantification of the number of apoptotic cells in the CA1 region of the hippocampus. Means ± SEM; n=7 mice per group. Two-tailed unpaired Student's t-test, t=1.542, p=0.15. **e-f.** Western blot analysis of the pro-apoptotic Bax (**e**) and anti-apoptotic Bcl2 (**f**) protein expression. Means ± SEM; n=6-8 mice per group. Two-tailed unpaired Student's t-test, t=1.515, p=0.155, Bax; t=0.39, p=0.705, Bcl2. **g.** FACS quantification CD45 high-CD11b+ cells from the CNS of n-3 deficient and n-3 sufficient mice. Means ± SEM; n=4 mice per group Two-tailed unpaired Student's t-test, t=0.51, p=0.63. **h-m.** GFAP (**h**), IgG (**j**) and Claudin-5 (**l**) immunoreactivity in n-3 sufficient and n-3 deficient mice. Means ± SEM; n=3-5 mice per group. Two-tailed unpaired Student's t-test, t=0.18, p= 0.86, GFAP; t=1.364, p=0.215, IgG; t=1.718, p= 0.146, Claudin-5. **i,k,m.** Representative images of GFAP (**i**), IgG (**k**) and Claudin-5 (Scale bar=100µM) (**m**) immunostainings (2 independent experiments). Arrows highlight claudin-5 staining. Source data are provided as a Source Data file.

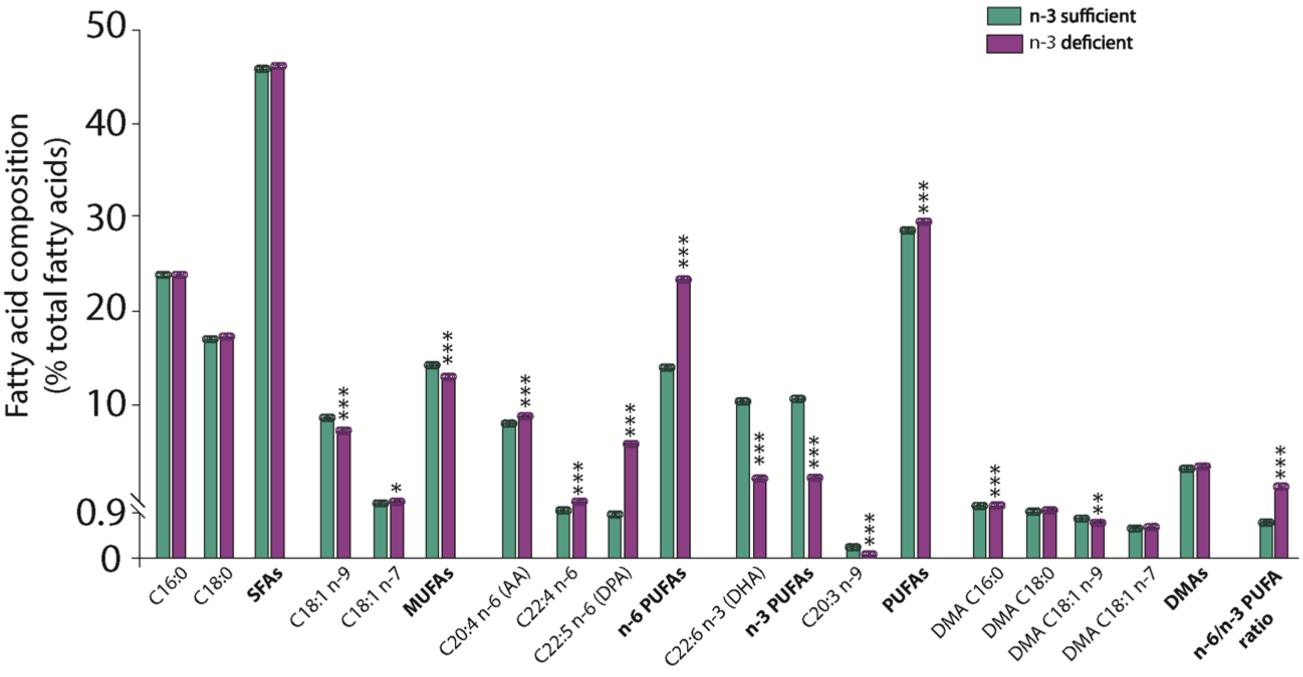

Supplementary Figure 4. Maternal n-3 PUFA deficiency modulates brain lipid composition. Fatty acid composition of the cortex varies according to maternal n-3 PUFA intake. Means ± SEM; n=6 mice per group. Two-tailed unpaired Student's t-test; t=11.94, -***p<0.0001, C18:1n-9; t=3.154, *p=0.01, C18:1n-7; t=7.27, ***p<0.0001, MUFAs; t=4.643, ***p=0.0009, AA; t=21.77, ***p<0.0001, C22:4n-6; t=36.09, ***p<0.0001, DPA n-6; t=35.78, ***p<0.0001, n-6 PUFAs; t=39.03, ***p<0.0001, DHA; t=39.36, ***p<0.0001, n-3 PUFAs; t=12.65, ***p<0.0001, C20:3n-9; t=5.371, ***p=0.0003, PUFAs; t=4.635, ***p=0.0009, Dimethylacetals (DMA) C16:0; t=4.271, **p=0.0016, DMA C18:1n-9; t=34.44, ***p<0.0001 n-6/n-3 ratio. Source data are provided as a Source Data file.

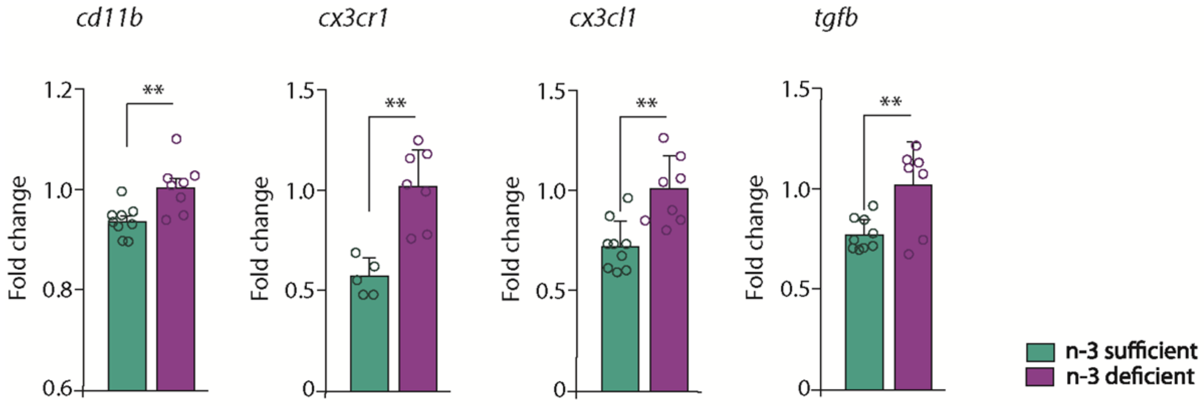

Supplementary Figure 5. Maternal n-3 PUFA deficiency enhances the expression of complement protein C3 and of genes involved in microglia-mediated synaptic refinement. *cd11b*, *cx3cr1*, *cx3cl1* and *tgfb* mRNA expression is increased in the hippocampus of n-3 deficient mice. Means ± SEM; n=5-9 mice per group. Two-tailed unpaired Student's t-test; t=3.115, **p=0.0076, *cd11b*; t=4.58, **p=0.001, *cx3cr1*; t=3.916, **p=0.0016, *cx3cl1*; t=3.184, **p=0.0066, *tgfb*. Source data are provided as a Source Data file.

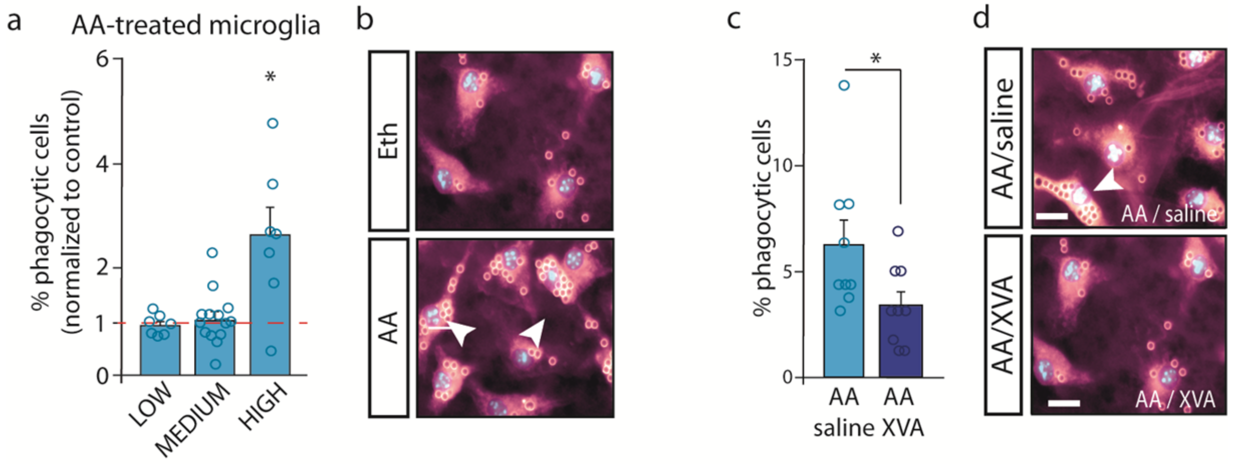

Supplementary Figure 6. a. Quantification of the percentage of microglial phagocytic cells 24h after application of AA. Means ± SEM; n=7-8 experiments per condition. Two-tailed unpaired Student's t-test, Eth vs AA, t=0.543, p=0.596, LOW; t=0.134, p=0.89, MEDIUM; t=2.87, *p=0.013, HIGH. **b.** Representative images of microglial cell in primary culture phagocyting latex beads from 3 independent experiments. Scale bar=10µm. Arrows: highly phagocytic cells (>10 beads per cell body). **c.** Application of the CR3 antagonist XVA-143 significantly reduces the phagocytic activity of microglia towards beads. Means ± SEM; n=9 experiments per condition. Two-tailed unpaired Student's t-test; t=2.216, *p=0.042. **d.** Representative images of AA/saline- and AA/XVA-treated microglial cells in primary culture from 3 independent experiments. Scale bar=10µm. Arrowhead: highly phagocytic microglia. Source data are provided as a Source Data file.

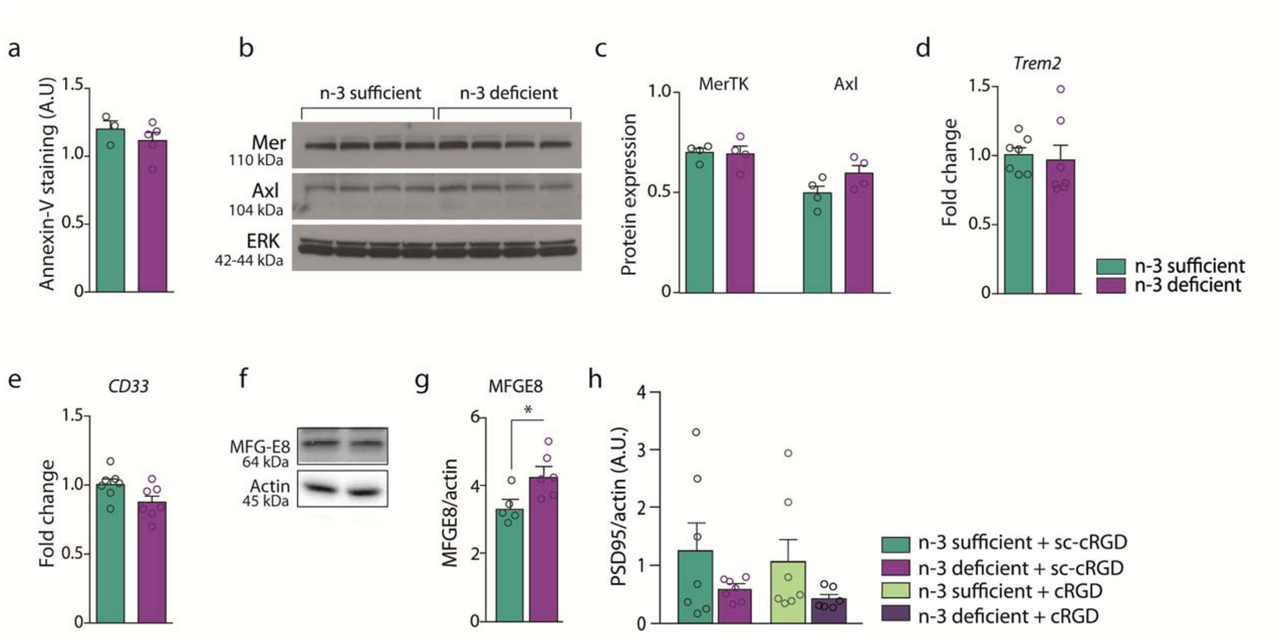

Supplementary Figure 7. Maternal n-3 PUFA deficiency enhances microglial phagocytic capacity in a PS recognition-independent manner. a. Annexin V staining is not different between n-3 deficient and n-3 sufficient mice. Means ± SEM; n=3-5 mice per group. Two-tailed unpaired Student's t-test; t=0.911, p= 0.397. **b.** Representative Western blots for Mer and Axl proteins from 2 independent experiments. **c.** Quantification of Mer and Axl protein in -3 deficient and n-3 sufficient mice. Means ± SEM; n=4 mice per group. Two-tailed unpaired Student's t-test; t=0.169, p=0.87, Mer; t=1.824, p=0.12, Axl. **d-e.** RT-qPCR detects the relative abundance of *trem2* (**d**) and *cd33* (**e**) mRNA in hippocampus of n-3 deficient and n-3 sufficient mice. Means ± SEM; n=7 mice per group. Two-tailed unpaired Student's t-test; t=0.329, p=0.75, *trem2*; t=2.158, p=0.052, *cd33*. **f.** Representative Western blots for MFG-E8 protein from 2 independent experiments. **g.** Quantification of MFG-E8 protein level in n-3 deficient vs n-3 sufficient mice. Means ± SEM; n=5-6 mice. Two-tailed unpaired Student's t-test; t=2.648, *p=0.0266. **h.** Quantification of PSD95 protein expression in n-3 sufficient *vs* n-3 deficient mice treated with cRGD or its control (scrambled peptide sc-cRGD). Means ± SEM; n=6-7 mice per group. Two-way ANOVA: diet effect, F(1,23)=4.017, p=0.057; treatment effect, F(1,23)=0.3417, p=0.56; interaction, F(1,23)=1.24x10^{-5}, p=0.99. Source data are provided as a Source Data file.

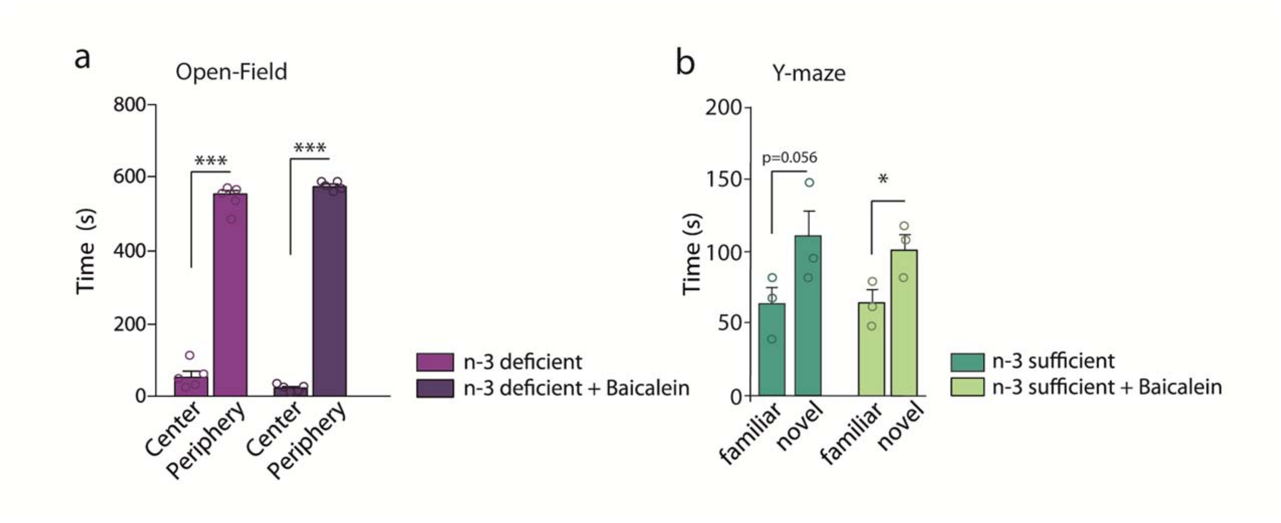

Supplementary Figure 8. Inhibition of the 12/15 LOX enzyme with baicalein does not affect the behavior of mice in the open Field. a. Time spent in the center *vs* periphery of the Open-Field. Means ± SEM; n=5 mice per group. Paired t-test: n-3 deficient group, ***p<0.0001; n-3 deficient + baicalein group, ***p<0.0001. **b.** Time spent in novel *vs* familiar arm in the Y maze task in P21 n-3 sufficient mice treated with baicalein or its vehicle. Means ± SEM; n=3 mice per group. Two-tailed paired t-test: n-3 sufficient group, p=0.056; n-3 sufficient + baicalein group, *p=0.038. Source data are provided as a Source Data file.

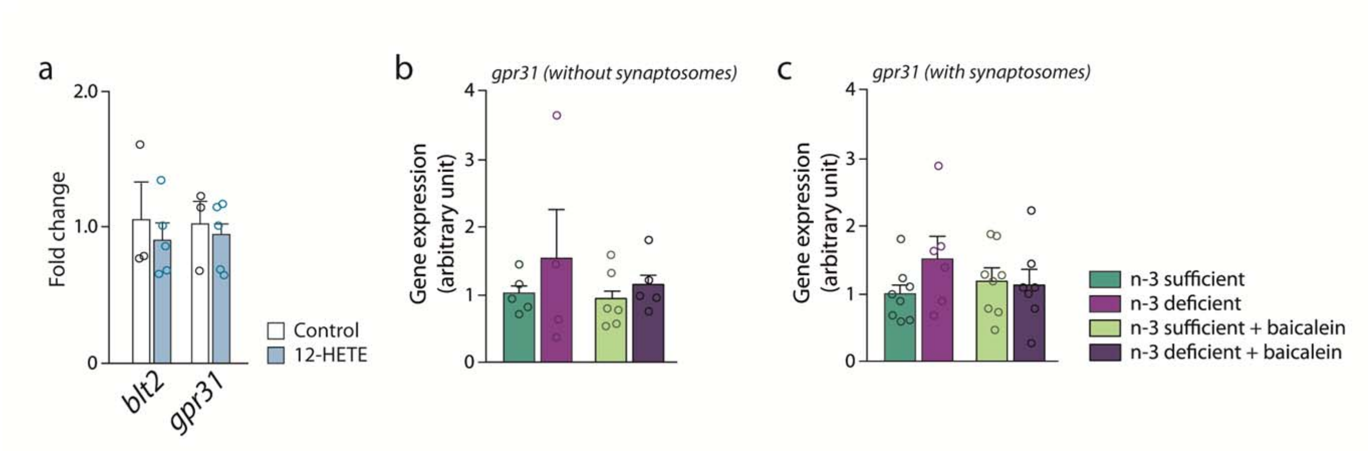

Supplementary Figure 9. The 12/15-LOX/12-HETE signaling pathway-induced microglial phagocytosis is independent from a modulation of the 12-HETE receptors expression both *in vitro* and *ex vivo*. a. mRNA expression of the two principal 12-HETE receptors BLT2 and GPR31 in primary microglia culture. Means ± SEM; n=3-5 experiments per condition. Two-tailed unpaired Student's t-test, *blt2*: vehicle vs 12-HETE, t=0.56, p=0.596, *gpr31*: vehicle vs 12-HETE t=0.52, p=0.62. **b.** Quantification of *gpr31* mRNA expression in freshly sorted n-3 deficient and n-3 sufficient microglia, treated with baicalein or its vehicle. Means ± SEM; n=4-6 per group. Two-way ANOVA: diet effect, F(1,15)=1.02, p=0.33; treatment effect, F(1,15)=0.51, p=0.49; interaction, F(1,15)=0.19, p=0.67. **c.** Quantification of *gpr31* mRNA expression in freshly sorted n-3 deficient and n-3 sufficient microglia, exposed to synaptosomes and treated with baicalein or its vehicle. Means ± SEM; n=6-8 per group. Two-way ANOVA: diet effect, F(1,25)=1.27, p=0.279; treatment effect, F(1,25)=0.63, **p=0.0028; interaction, F(1,25)=1.95, p=0.17. Source data are provided as a Source Data file.
