# Supplementary material for: Essential omega-3 fatty acids tune microglial phagocytosis of synaptic elements in the mouse developing brain
Source: Nat Commun. 2020 Nov 30;11:6133. doi: 10.1038/s41467-020-19861-z (PMC7704669; doi:10.1038/s41467-020-19861-z)
Supplement: Supplementary file 3 — Reporting Summary [file 41467_2020_19861_MOESM3_ESM.pdf]

nature research

Corresponding author(s): Agnes Nadjar

Last updated by author(s): Oct 1, 2020

Reporting Summary

Nature Research wishes to improve the reproducibility of the work that we publish. This form provides structure for consistency and transparency in reporting. For further information on Nature Research policies, see Authors & Referees and the Editorial Policy Checklist .

Statistics

For all statistical analyses, confirm that the following items are present in the figure legend, table legend, main text, or Methods section.

n/a	Confirmed	
☐	☒	The exact sample size (*n*) for each experimental group/condition, given as a discrete number and unit of measurement
☒	☐	A statement on whether measurements were taken from distinct samples or whether the same sample was measured repeatedly
☐	☒	The statistical test(s) used AND whether they are one- or two-sided<br>*Only common tests should be described solely by name; describe more complex techniques in the Methods section.*
☐	☒	A description of all covariates tested
☐	☒	A description of any assumptions or corrections, such as tests of normality and adjustment for multiple comparisons
☐	☒	A full description of the statistical parameters including central tendency (e.g. means) or other basic estimates (e.g. regression coefficient) AND variation (e.g. standard deviation) or associated estimates of uncertainty (e.g. confidence intervals)
☐	☒	For null hypothesis testing, the test statistic (e.g. *F*, *t*, *r*) with confidence intervals, effect sizes, degrees of freedom and *P* value noted<br>*Give P values as exact values whenever suitable.*
☒	☐	For Bayesian analysis, information on the choice of priors and Markov chain Monte Carlo settings
☒	☐	For hierarchical and complex designs, identification of the appropriate level for tests and full reporting of outcomes
☐	☒	Estimates of effect sizes (e.g. Cohen's *d*, Pearson's *r*), indicating how they were calculated

Our web collection on statistics for biologists contains articles on many of the points above.

Software and code

Policy information about availability of computer code

Data collection	Image J (Simple Neurite Tracer plugin) 1.52d<br>Fiji (3D object counter plugin) 1.48c<br>Mercator Pro 7.9.11<br>NIS Elements AR 3.26<br>Wallac 1420 instrument Workout 2.5 (PerkinElmer)<br>AMT Image Capture Engine Software 601.384<br>Diva 8.0 (BD Biosciences)<br>FlowJo 10.5 (FlowJo, LLC)<br>Nanostring nCounter® System: nCounter Prep Station and nCounter Digital Analyzer (Version 2.1.2.3)<br>LightCycler®480 software release 1.5.0 SP4<br>MetaMorph software version 7<br>GeneTools software (Synoptics, Cambridge, UK) 2.0<br>Smart (Panlab, Barcelona, Spain) 3.0
Data analysis	Miru (formerly known as BioLayout Express3D) ® Kajeka 2014-16 https://kajeka.com/graphia-professional/<br>GraphPad Prism 7.0<br>Nanostring nSolver™ Analysis Software (version 2.0)<br>Markov clustering algorithm

For manuscripts utilizing custom algorithms or software that are central to the research but not yet described in published literature, software must be made available to editors/reviewers. We strongly encourage code deposition in a community repository (e.g. GitHub). See the Nature Research guidelines for submitting code & software for further information.

Data

Policy information about availability of data

All manuscripts must include a data availability statement. This statement should provide the following information, where applicable:

- Accession codes, unique identifiers, or web links for publicly available datasets
- A list of figures that have associated raw data
- A description of any restrictions on data availability

Source data are provided with this paper. All relevant data are available from the authors upon request. Fatty acids derivatives data are available on Metabolights. Our study is identified as MTBLS1952. Transcriptomic data are available in the GEO database. Accession number: GSE158181.

Field-specific reporting

Please select the one below that is the best fit for your research. If you are not sure, read the appropriate sections before making your selection.

- [x] Life sciences
- [ ] Behavioural & social sciences
- [ ] Ecological, evolutionary & environmental sciences

For a reference copy of the document with all sections, see nature.com/documents/nr-reporting-summary-flat.pdf

Life sciences study design

All studies must disclose on these points even when the disclosure is negative.

Sample size

S
We did not use a mathematical method for sample size calculation. We have conducted experiments along lines that are classically used in the literature and are based on previous/pilot experiments ran in the lab over the last 15+ years, and that have provided results with statistical significance. On average, we used (after removal of the outliers, see below for the calculation of outliers): N=5 for Golgi staining experiments; N=5-8 for Western blotting; N=5-10 for immunostaining; N=10-17 for Y-maze; N=4 for lipidomics; N=7-8 for ELISA; N=3-20 for ex-vivo phagocytosis; N=4-6 for transcriptomics; N=5-14 (each N containing duplicates or triplicates for each measurement) for in vitro studies; N=3 for electron microscopy (>600 measurements over the 3 animals). We respected the 3R rules, by not unnecessarily increasing the numbers of animal used in our experiments.

Data exclusions

We pre-established mean+/- 2SD as exclusion threshold (outlier). On this basis, we excluded data in experiments presented in Figure 1D (cofilin WB, 1 n-3 deficient mouse), 1E (Y maze, 1 n-3 sufficient mouse); Figure 4A (IHC C1q, 1 n-3 deficient mouse), 4B (IHC CD11b, 1 n-3 deficient mouse), 4D (ELISA C3, 1 n-3 sufficient and 1 n-3 deficient mice), 4F (Y-maze/XVA143, 1 n-3 sufficient mouse); Figure 6F (ex vivo phagocytosis + baicalein, 1 n-3 deficient microglia); Figure 7C (ddPCR C3aR without synaptosome, 1 n-3 deficient mouse); Supplementary Figure 3F (WB Bcl2, 1n-3 deficient and 1 n-3 sufficient mouse); Supplementary Figure 5 (CX3CR1 qPCR, 1 n-3 sufficient mouse); Supplementary Figure 7G (WB MFGE8, 1 n-3 sufficient and 2 n-3 deficient mouse)

Replication

All experiments were successfully replicated at least once.

Randomization

All samples/animals were randomized between groups

Blinding

All experiments were performed and analyzed by investigators blinded to group identification

Reporting for specific materials, systems and methods

We require information from authors about some types of materials, experimental systems and methods used in many studies. Here, indicate whether each material, system or method listed is relevant to your study. If you are not sure if a list item applies to your research, read the appropriate section before selecting a response.

Materials & experimental systems

n/a	Involved in the study	
[ ]	[x]	Antibodies
[x]	[ ]	Eukaryotic cell lines
[x]	[ ]	Palaeontology
[ ]	[x]	Animals and other organisms
[x]	[ ]	Human research participants
[x]	[ ]	Clinical data

Methods

n/a	Involved in the study	
[x]	[ ]	ChIP-seq
[ ]	[x]	Flow cytometry
[x]	[ ]	MRI-based neuroimaging

Antibodies

Antibodies used

anti-Iba1 Rabbit WAKO 019-19741
anti-PSD95 Rabbit D27E11 Cell Signaling Technology 3450S

anti-PSD95 mouse K28-43 anticorps-en-ligne.fr ABIN1304920
anti-CD11b rat 5C6 Biorad (formerly AbD Serotec) MCA711
anti-C1q Rabbit Monoclonal 4.8 Abcam ab182451
anti-C3 Rabbit polyclonal Dako / Agilent A006302 AB_578478
anti-C3aR rat 14D4 Hycult biotech HM1123
anti-GFAP Rabbit polyclonal Dako Z03334
anti-NeuN mouse A60 Millipore MAB377
anti-Annexin V rabbit polyclonal Abcam ab14196
anti-claudin 5 Mouse Clone 4C3C2 Life Technologies: Invitrogen 35-2500
IRDye 800-conjugated anti-mouse IgG (H+L) Goat polyclonal Rockland antibodies & assays 610-145-121
anti-mouse Alexa fluor 594 Goat polyclonal invitrogen A-11005
anti-rabbit Alexa fluor 488 Donkey polyclonal invitrogen A-21206
APC anti-CD11b rat M1/70 eBioscience 17-0112-82
FITC anti-CD11b rat M1/70 BDBiosciences 553310
PerCP-Cy™5.5 Anti-Mouse CD45 rat 30-F11 BDBiosciences 550994
APC Anti-Ly6G rat 1A8 Biolegend 127614
APC-Cy™7 Anti-Mouse Ly-6C rat AL-21 BDBiosciences 560596
PE anti-rat goat polyclonal BDBiosciences 550767
V450 anti-CD11b rat M1/70 BDBiosciences 560455
unconjugated anti-FCRLS Rat Clone 4G11 custom
anti-Bax Rabbit N-20 Santa Cruz Biotechnology SC-493
anti-Bcl2 Rabbit N-19 Santa Cruz Biotechnology SC-492
anti-Mer Goat polyclonal R&D systems AF591
anti-Axl Goat polyclonal R&D systems AF854
anti-MFG-E8 Goat polyclonal R&D systems AF2805
anti-GluA1 Rabbit polyclonal Santa Cruz Biotechnology SC-28799
anti-GluA2 Goat polyclonal Santa Cruz Biotechnology SC-7611
anti-GluN2A Goat polyclonal Santa Cruz Biotechnology SC-1468
anti-GluN2B Goat polyclonal Santa Cruz Biotechnology SC-1469
anti-GluN1 Goat polyclonal Santa Cruz Biotechnology SC-31556
anti-SAP102 Rabbit polyclonal Synaptic System 124213
anti-cofilin mouse monoclonal Abcam Ab54532
anti-actin Rabbit 13E5 Cell Signaling Technology 4970S
anti-GAPDH Rabbit D16H11 Cell Signaling Technology 5174S
anti-ERK1/2 Rabbit monoclonal Cell Signaling Technology 137F5
Peroxidase AffiniPure anti-rabbit IgG (H+L) Donkey polyclonal Jackson ImmunoResearch 711-035-152
Peroxidase AffiniPure anti-mouse IgG (H+L) Donkey polyclonal Jackson ImmunoResearch 715-035-151
biotinylated anti-rabbit IgG (H+L) Goat polyclonal Vector BA-1000
biotinylated conjugated anti-rat IgG (H+L) rabbit Vector BA4001

Validation

Controls were performed by omitting the primary Ab for IHC or directly-conjugated antibody for FACS sorting. All the Ab used in our study have been validated by others.
- anti-Iba1 Rabbit WAKO 019-19741 IHC https://labchem-wako.fujifilm.com/us/product/detail/W01W0101-1974.html
- anti-PSD95 Rabbit D27E11 Cell Signaling Technology 3450S WB PSD95 (D27E11) XP® Rabbit mAb detects endogenous levels of total PSD95 protein. https://www.cellsignal.com/products/primary-antibodies/psd95-d27e11-xp-rabbit-mab/3450?Ntk=Products&Ntt=3450
- anti-PSD95 mouse K28-43 anticorps-en-ligne.fr ABIN1304920 IHC https://www.anticorps-enligne.fr/antibody/1304920/anti-Discs,+Large+Homolog+4+Drosophila+DLG4+antibody/
- anti-CD11b rat 5C6 Biorad (formerly AbD Serotec) MCA711 IHC This product has been reported to work in the following applications. This information is derived from testing within our laboratories, peer-reviewed publications or personal communications from the originators. Please refer to references indicated for further information. https://www.bio-rad-antibodies.com/monoclonal/mouse-cd11b-antibody-5c6-mca711.html?f=purified#references
- anti-C1q Rabbit Monoclonal 4.8 Abcam ab182451 IHC Knockout validated https://www.abcam.com/c1q-antibody-48-ab182451.html
- anti-C3 Rabbit polyclonal Dako / Agilent A006302 AB_578478 ELISA
- anti-C3aR rat 14D4 Hycult biotech HM1123 IHC For immunohistochemistry and flow cytometry, dilutions to be used depend on detection system applied. Kiafard Z, et al; Use of monoclonal antibodies to assess expression of anaphylatoxin receptors in tubular epithelial cells of human, murine and rat kidneys. Immunobiology 2007, 212: 129.
- anti-GFAP Rabbit polyclonal Dako Z03334 IHC PMID: 25309435
- anti-NeuN mouse A60 Millipore MAB377 IHC Anti-NeuN Antibody, clone A60 detects level of NeuN and has been published and validated for use in FC, IC, IF, IH, IH(P), IP and WB. https://www.merckmillipore.com/FR/fr/product/Anti-NeuN-Antibody-clone-A60,MM_NF-MAB377#anchor_REF
- anti-Annexin V rabbit polyclonal Abcam ab14196 WB Our Abpromise guarantee covers the use of ab14196 in the following tested applications. https://www.abcam.com/annexin-vanxa5-antibody-ab14196.html
- anti-claudin 5 Mouse Clone 4C3C2 Life Technologies: Invitrogen 35-2500 IHC This Antibody was verified by Relative expression to ensure that the antibody binds to the antigen stated. View Details https://www.thermofisher.com/antibody/product/Claudin-5-Antibody-clone-4C3C2-Monoclonal/35-2500

- IRDye 800-conjugated anti-mouse IgG (H+L) Goat polyclonal Rockland antibodies & assays 610-145-121 IHC https://rockland-inc.com/store/DyLight-Conjugated-Antibodies-610-145-121-O4L_8300.aspx
- anti-mouse Alexa fluor 594 Goat polyclonal invitrogen A-11005 IHC https://www.thermofisher.com/antibody/product/Goat--anti-Mouse-IgG-H-L-Cross-Adsorbed-Secondary-Antibody-Polyclonal/A-11005
- anti-rabbit Alexa fluor 488 Donkey polyclonal invitrogen A-21206 IHC https://www.thermofisher.com/antibody/product/Donkey-anti-Rabbit-IgG-H-L-Highly-Cross-Adsorbed-Secondary-Antibody-Polyclonal/A-21206
- APC anti-CD11b rat M1/70 eBioscience 17-0112-82 FACS
- FITC anti-CD11b rat M1/70 BDBiosciences 553310 FACS
- PerCP-Cy™5.5 Anti-Mouse CD45 rat 30-F11 BDBiosciences 550994 FACS
- APC Anti-Ly6G rat 1A8 Biolegend 127614 FACS
- APC-Cy™7 Anti-Mouse Ly-6C rat AL-21 BDBiosciences 560596 FACS
- PE anti-rat goat polyclonal BDBiosciences 550767 FACS
- V450 anti-CD11b rat M1/70 BDBiosciences 560455 FACS
- unconjugated anti-FCRLS Rat Clone 4G11 custom FACS Butovsky et al 2014 Krasemann et al 2017
- anti-Bax Rabbit N-20 Santa Cruz Biotechnology SC-493 WB Suitable for use as control antibody for Bax siRNA (h): sc-29212, Bax siRNA (m): sc-29213, Bax shRNA Plasmid (h): sc-29212-SH, Bax shRNA, Plasmid (m): sc-29213-SH, Bax shRNA (h) Lentiviral Particles: sc-29212-V and Bax shRNA (m) Lentiviral Particles: sc-29213-V. PMID: 27448980 , 27030982, 27488203, 26542803
- anti-Bcl2 Rabbit N-19 Santa Cruz Biotechnology SC-492 WB Suitable for use as control antibody for Bcl-2 siRNA (h): sc-29214, Bcl-2 siRNA (m): sc-29215, Bcl-2 shRNA Plasmid (h): sc-29214-SH, Bcl-2 shRNA, Plasmid (m): sc-29215-SH, Bcl-2 shRNA (h) Lentiviral Particles: sc-29214-V and Bcl-2 shRNA (m) Lentiviral Particles: sc-29215-V. PMID: 30146791, 28760656, 29287688, 27821721, 27151590
- anti-Mer Goat polyclonal R&D systems AF591 WB "Fourgeaud L, Traves P, Tufail Y, Leal Bailey H, Lew E, Burrola P, et al. TAM receptors regulate multiple features of microglial physiology. Nature. 2016;532:240-244 1. Nagata, K. et al. (1996) J. Biol. Chem. 22:30022. 2. Crosier, K.E. and P.S Crosier (1997) Pathology 29:131."
- anti-Axl Goat polyclonal R&D systems AF854 WB "Fourgeaud L, Traves P, Tufail Y, Leal Bailey H, Lew E, Burrola P, et al. TAM receptors regulate multiple features of microglial physiology. Nature. 2016;532:240-244 "
- anti-MFG-E8 Goat polyclonal R&D systems AF2805 WB https://www.rndsystems.com/products/mouse-mfg-e8-antibody_af2805
- anti-GluA1 Rabbit polyclonal Santa Cruz Biotechnology SC-28799 WB Schiffer, H.H., et al. 1997. Rat GluR7 and a carboxy-terminal splice variant, GluR7β, are functional kainate receptor subunits with a low sensitivity to glutamate. Neuron 19: 1141-1146.
- anti-GluA2 Goat polyclonal Santa Cruz Biotechnology SC-7611 WB Schiffer, H.H., et al. 1997. Rat GluR7 and a carboxy-terminal splice variant, GluR7β, are functional kainate receptor subunits with a low sensitivity to glutamate. Neuron 19: 1141-1146.
- anti-GluN2A Goat polyclonal Santa Cruz Biotechnology SC-1468 WB PMID: 28375208, 25220981
- anti-GluN2B Goat polyclonal Santa Cruz Biotechnology SC-1469 WB PMID: 28375208, 25220981
- anti-GluN1 Goat polyclonal Santa Cruz Biotechnology SC-31556 WB Schiffer, H.H., et al. 1997. Rat GluR7 and a carboxy-terminal splice variant, GluR7β, are functional kainate receptor subunits with a low sensitivity to glutamate. Neuron 19: 1141-1146.
- anti-SAP102 Rabbit polyclonal Synaptic System 124213 WB 124 213 WB; KO verified; tested species: mouse Zhu F, Cizeron M, Qiu Z, Benavides-Piccione R, Kopanitsa MV, Skene NG, Koniaris B, DeFelipe J, Fransén E, Komiyama NH, Grant SGN, et al. Neuron 2018
- anti-cofilin mouse monoclonal Abcam Ab54532 WB Our Abpromise guarantee covers the use of ab54532 in the following tested applications. Ryu HG et al. Upf1 regulates neurite outgrowth and branching by transcriptional and post-transcriptional modulation of Arc. J Cell Sci 132:N/A (2019).
- anti-actin Rabbit 13E5 Cell Signaling Technology 4970S WB Antibody Guarantee https://www.cellsignal.com/products/primary-antibodies/b-actin-antibody/4967?Ntk=Products&Ntt=4967
- anti-GAPDH Rabbit D16H11 Cell Signaling Technology 5174S WB Antibody Guarantee https://www.cellsignal.com/products/primary-antibodies/gapdh-14c10-rabbit-mab/2118?Ntk=Products&site-search-type=Products&N=4294956287&Ntt=+2118s&fromPage=plp&_requestid=671801
- anti-ERK1/2 Rabbit monoclonal Cell Signaling Technology 137F5 WB Antibody Guarantee https://www.cellsignal.com/products/primary-antibodies/p44-42-mapk-erk1-2-137f5-rabbit-mab/4695?Ntk=Products&site-search-type=Products&N=4294956287&Ntt=137f5&fromPage=plp
- Peroxidase AffiniPure anti-rabbit IgG (H+L) Donkey polyclonal Jackson ImmunoResearch 711-035-152 WB
- Peroxidase AffiniPure anti-mouse IgG (H+L) Donkey polyclonal Jackson ImmunoResearch 715-035-151 WB
- biotinylated anti-rabbit IgG (H+L) Goat polyclonal Vector BA-1000 IHC
-biotinylated conjugated anti-rat IgG (H+L) rabbit Vector BA4001 IHC

Animals and other organisms

Policy information about studies involving animals; ARRIVE guidelines recommended for reporting animal research

Laboratory animals	CD1 mice, males and females; Pups were studied at P21. We also used adult mice for mating purposes (3-5 mo).
Wild animals	No wild animals were used in the study.
Field-collected samples	No field collected samples were used in the study.
Ethics oversight	Animal experiments were carried out according to the Quality Reference System of INRA and approved by the local ethical committee of the University of Bordeaux for care and use of animals (#APAFIS 4198 and 15533).

Note that full information on the approval of the study protocol must also be provided in the manuscript.

Flow Cytometry

Plots

Confirm that:

- [x] The axis labels state the marker and fluorochrome used (e.g. CD4-FITC).
- [x] The axis scales are clearly visible. Include numbers along axes only for bottom left plot of group (a 'group' is an analysis of identical markers).
- [x] All plots are contour plots with outliers or pseudocolor plots.
- [x] A numerical value for number of cells or percentage (with statistics) is provided.

Methodology

Sample preparation	Brains were homogenized in Hanks' Balanced Salt Solution (HBSS), pH 7.4 passing through a 70 μm nylon cell strainer. Homogenates were centrifuged at 600g for 6 min. Supernatants were removed and cell pellets were re-suspended in 70% isotonic Percoll (GE-Healthcare, Aulnay sous Bois, France). A discontinuous Percoll density gradient was set up as follows: 70%, 35% and 0% isotonic Percoll. Gradients were centrifuged at 2000g for 20min. Microglia cells were collected at the interphase between the 70% and 35% Percoll layers. Cells were washed and stained before analyses or sorting.
Instrument	FACS data were acquired using an LSR Fortessa 2-Blue 6-Violet 3-Red 5-YelGr laser configuration (BD Biosciences). Cells were sorted using a FACS Aria 5-Blue 2-Violet 2-Red laser configuration (BD Biosciences).
Software	Flow cytometry analysis were performed using Diva 8 (BD Biosciences) and FlowJo 10.5 (FlowJo, LLC) softwares
Cell population abundance	For each brain extraction approximately 3×10 5 microglia cells were isolated
Gating strategy	Gating strategy for ex vivo microglia phagocytosis experiments corresponding to Figure 2, 3 and 7. Figure shows representative FACS plots of gates P1 (Cells), P2 (Single cells), P3 (Ly6G-ve/Ly6G-ve to gate out neutrophil and monocyte populations), P4 (CD45low/CD11b+ve Microglia), P5 (pHrodo +ve synaptosomes phagocytosed by microglia).

- [x] Tick this box to confirm that a figure exemplifying the gating strategy is provided in the Supplementary Information.
